# Electroplating of Multiple Materials in Parallel Using Patterned Gels with Applications in Electrochemical Sensing

**DOI:** 10.3390/s20030886

**Published:** 2020-02-07

**Authors:** Aliakbar Mohammadzadeh, Alison Fox-Robichaud, P. Ravi Selvaganapathy

**Affiliations:** 1 Department of Mechanical Engineering, McMaster University, Hamilton, ON L8S 4L7, Canada; 2 Department of Medicine, McMaster University, Hamilton, ON L8L 2X2, Canada; afoxrob@mcmaster.ca

**Keywords:** electrodeposition, gel printing, parallel electroplating, microTAS, microfluidics, electrochemical sensors

## Abstract

Electrodeposition is a versatile technique for the fabrication of electrodes in micro-electroanalytical devices. Conductive but low-cost materials, such as copper, can be coated with functional yet higher-cost materials such as gold or silver using electrodeposition to lower the overall cost while maintaining functionality. When the electrodeposition of multiple materials is required, current methods use a multistep process that deposits one material at a time, which requires a significant amount of time and a significant number of steps. Additionally, they use a large volume of electrolytes suitable for coating large objects, which is wasteful and unnecessary for the prototyping or coating of microelectrodes with a small area. In this paper, a new method of electroplating is introduced in which we used gels to immobilize and pattern electroplating electrolytes on a substrate surface. Agarose, as an immobilizing medium, enables the immersion of the substrate in a common working electrolyte without cross-mixing different electrolytes. We demonstrate the printing of jelly electrolytes by using spot-dispensing or microfluidic flow. Xurographically patterned films laminated on the substrate function as a mask and confine the printed gels to desired locations. After printing, the substrate is placed in a common working electrolyte container, and multimaterial patterns are produced through the application of an electrical current in a single step.

## 1. Introduction

Micro-total analysis systems (µTAS) have been the focus of an extensive amount of research over the past three decades [1]. Applications of such systems include, but are not limited to, point of care medical diagnostics [2,3], drug delivery [4], and environmental monitoring [5,6]. In these analytical systems, electrodes, along with microchannel networks, are integrated for different functionalities such as actuation, heating, or sensing [7]. In sensor devices, microelectrodes that are mostly made of high-cost materials such as gold and silver are fabricated to perform electrochemical operations. Techniques for the fabrication of these electrodes are mainly sourced from the integrated circuit industry and require complicated, tedious, and multiple-step processes.

In the fabrication of microanalytical devices, electrodes are commonly fabricated by physical vapor deposition (PVD) or through the sputtering of noble metals [8,9,10,11]. These methods use photolithography to pattern electrode structures. In addition, high-vacuum conditions are needed to sputter and deposit noble metal layers on a substrate. Since these methods are derived from the electronics industry, they are suitable for the deposition of one material at a time. Therefore, for the deposition of multiple materials, repeated deposition and photolithography steps are required that make the entire process complicated and expensive.

Since electrochemical deposition does not require costly facilities, this technique has been used increasingly in recent years for the fabrication of electrodes in microanalytical devices [12,13]. The electrical and mechanical properties of electrodes made by electrodeposition are well suited for this application. However, microanalytical devices usually require electrodes made of multiple materials. The sequential process of depositing/removing one material/mask makes the entire multistep process long and complicated in terms of the deposition of multiple materials. Although the consumption of a large volume of electrolytes is reasonable when coating electrodes on a large scale, it is wasteful for small-area electrodes. In order to overcome these limitations, inkjet and screen printing techniques have been introduced [14].

Screen and inkjet printing are nonvacuum and low cost and have direct patterning techniques for the deposition of thin conductive films [15]. In these methods, conductive nanoparticles such as silver, gold, carbon, and platinum [16,17] are dissolved in the form of ink/paste and printed on a substrate by inkjet and screen printers. After deposition, the printed ink/paste is dried and sintered at a high temperature. A variety of microfluidic devices have been developed using these techniques, including heaters [18] and dielectrophoretic chips [19]. Despite the ease of operation and cost-effectiveness of this technique, there are significant issues associated with the mechanical properties of printed electrodes; for instance, they are easily cracked by thermal expansion or mechanical bonding [20]. In addition, the clogging of inkjet printer nozzles due to the use of nanoparticles is a frequent problem [21]. Additionally, these printed electrodes have less conductivity than do bulk materials [15]. 

A suitable technique that is able to integrate multiple functional materials while being cost-effective and fast is required for the fabrication of electrodes in these microanalytical devices. This technique needs to be performed in a noncleanroom environment without the use of costly high-vacuum equipment. Here, a novel and robust fabrication technique able to deposit layers of gold and silver in a single process and in parallel is introduced. With this technique, gold and silver electrolytes are mixed separately with agarose gel and printed on nickel-coated substrates. After printing, the substrate is immersed in a common working electrolyte and an electrical current is applied to electrodeposit layers of gold and silver. The entire process is carried out in a noncleanroom environment with low-cost facilities, such as a power supply and a cutting plotter. It is worth mentioning that although xurography (using a cutter plotter) has a lower resolution compared to other patterning techniques, such as photolithography, the resolution is sufficient for most microanalytical systems [22]. Accordingly, the base substrate and masks used in the process are fabricated through a xurography lamination technique, as described in our recent works [23,24]. In addition, using gel droplets enables us to electrodeposit different metals at the same time without cross-deposition. Further, using small amounts of solution as droplets minimizes the use of electrolytes, which is crucial for the rapid prototyping of microanalytical systems. We show the ability of this method by creating arrays in the form of dots and lines. In addition, we demonstrate its functionality through electrochemical sensing in a three-electrode system integrated into a microfluidic device. Finally, using gel droplets for electroplating has the potential for use in other additive manufacturing techniques, such as inkjet printers (fully automating the technique).

## 2. Methods

### 2.1. Base Electrode Fabrication

**Figure 1:**
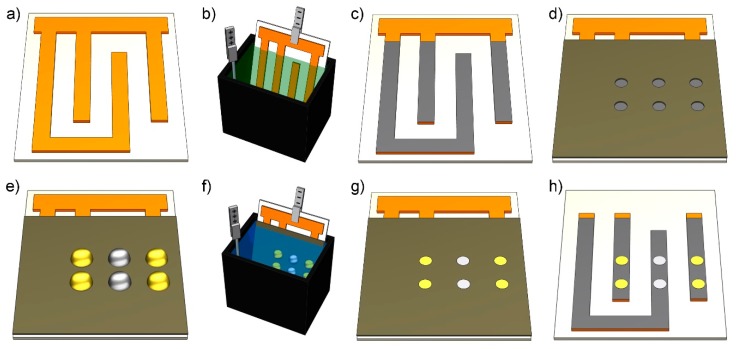
Parallel electroplating using printed gels. (**a**) Patterning of a copper polyimide foil laminated on a dicing tape; (**b**) nickel electrodeposition; (**c**) a nickel-coated substrate; (**d**) a substrate with a laminated mask; (**e**) a printing droplet of gold and silver electrolytes mixed with agarose gel; (**f**) electrodeposition in a 1-M potassium nitrate bath; (**g**) electrodeposited layers of gold and silver; and (**h**) detachment of the mask and cutting off of the contact pad to separate the electrodes.

The process of fabricating an array of base electrodes for electroplating was described in our previous work [24]. Briefly, a copper polyimide (PI) foil (Pyralux^®^, AC091200EV, DuPont, Wilmington, DE, USA) with an overall thickness of 21 µm (12 µm Cu and 9 µm PI) was patterned using a xurography technique in which a cutting plotter (FC-8600, Graphtec America Inc., Irvine, CA, USA) was used to create patterns. In addition, layers of dicing tape (1020 UV adhesive, Ultron Systems Inc., Moorpark, CA, USA) with a thickness of 95 µm each were used for improving the cut quality and developing patterns (peeling off unwanted regions of foil) (Figure 1a).

Next, a nickel electroplating bath consisting of 2 M of nickel (II) sulfate (NiSO_4_) (Sigma-Aldrich, St. Louis, MO, USA) and 0.5 M of boric acid (Caledon Laboratories Ltd., Georgetown, ON, Canada) with a pH of ~3–4 was prepared. Then, the base electrode layer was placed in the bath (Figure 1b) and a current density of 2 A dm^−2^ was used for five minutes to electrodeposit the nickel at room temperature (Figure 1c). Nickel, serving as a diffusion barrier layer, blocked the undesirable diffusion of copper into the electroplated layers [25]. Next, a layer of one-sided adhesive tape (9795R, 3M™, Saint Paul, MN, USA) with a 100-µm thickness (patterned with xurography and working as an electroplating mask) was aligned and laminated on top of the foil (Figure 1d).

### 2.2. Preparation of Gold and Silver Electroplating Bath Mixed with Agarose Gel

The gold electroplating bath was prepared by mixing 0.84 M sodium sulfite and sodium thiosulfate (Sigma-Aldrich, St. Louis, MO, USA). Then, 0.1 M gold chloride (Sigma-Aldrich, St. Louis, MO, USA) was added to the solution gradually in such a way that the final pH of the solution was about 7 [26]. For the silver electroplating bath, 30 g L^−1^ silver (as chloride) (Alfa Aesar, Tewksbury, MA, USA), 30 g L^−1^ potassium metabisulfite (Sigma-Aldrich, St. Louis, MO, USA), and 500 g L^−1^ sodium thiosulfate were mixed. The final pH of the solution was in the range of 4–5 [27]. After that, the solution was diluted with water (five-fold). Both of these plating solutions were chosen from noncyanide electrolytes due to safety and environmental considerations. The concentration of agarose (Bioshop Inc., Burlington, Canada) was 2 g L^−1^, and it was kept at 90 °C through placement in an oil bath. Finally, each electroplating solution was mixed with agarose separately in a ratio of 1:1. They were in two different containers at 90 °C.

### 2.3. Electroplating Procedure

Each mixture of electroplating electrolytes with agarose was printed manually by a micropipette in the form of droplets on the laminated mask (Figure 1e), but this can also be automated using an automated plotter. The laminated mask, which was previously patterned using xurography, confined the droplets to the exact location where they were originally printed. After that, the substrate was immersed in a common working electrolyte bath containing 1 M potassium nitrate (KNO_3_) (Sigma-Aldrich, St. Louis, MO, USA). A platinum wire electrode functioning as an anode was placed in the bath (Figure 1f). A source meter (2636, Keithley Instrument, Cleveland, OH, USA) was used to apply 675 mV. Electrodeposition was performed for 5 min at room temperature. Next, the substrate was ejected from the bath, rinsed with deionized water to remove the remaining gel solution on the substrate, and dried through nitrogen-purging (Figure 1g). The patterned electrodes were separated by cutting the contact pad. The electrode layer with the laminated mask can be integrated into a microfluidic system or used for other applications after detachment of the mask (Figure 1h).

## 3. Results and Discussion

### 3.1. Electroplating Using Printed Gels

**Figure 2:**
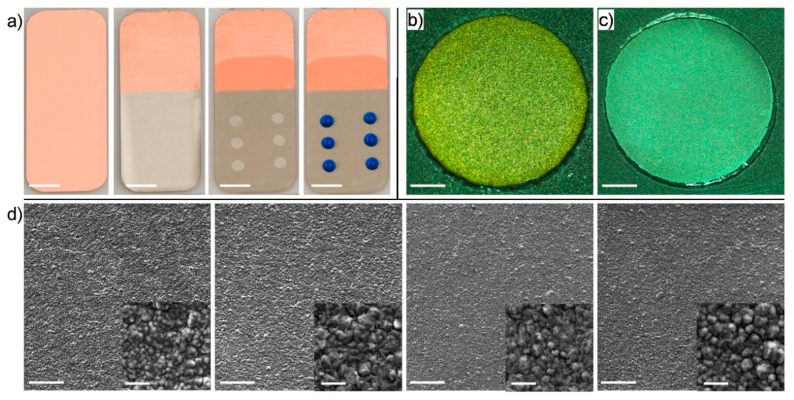
Electrodeposition of gold and silver through gel printing. (**a**) Substrate preparation, including nickel coating, mask lamination, and gel printing (scale bar = 7 mm); (**b**,**c**) electrodeposited layer of (b) gold and (c) silver (scale bar = 600 µm); (**d**) SEM images with insets of higher magnification for layers of Cu, Ni, Au, and Ag (from left to right) (scale bar = 100 µm; inset = 10 µm).

A single noble metal was first electroplated using printed gels. For this purpose, a base electrode made of copper polyimide foil 20 mm in length and 50 mm wide was fabricated using a xurography lamination technique. After that, nickel was coated on the electrode through electroplating. A mask patterned with xurography and consisting of holes with a 1.5-mm radius was laminated on the substrate. Next, a micropipette was used to print 10-µL droplets of gold electrolytes mixed on the electrode. The deposited droplets automatically self-aligned themselves to the patterned circular holes due to surface tension. The sequence of substrate preparation, including the initial copper foil, nickel-coating, mask lamination, and dispensing of the gels (gels were added with blue dye for visualization), is shown in Figure 2a. Next, an external direct current (DC) power supply was connected to the substrate. The substrate was immersed into a common working electrolyte container and electrodeposited. Figure 2b shows a microscope image of the electrodeposited layers of gold on nickel. Silver was electroplated using printed gel by carrying out the same experiment, but with silver plating salts mixed with the agarose. Figure 2c shows the electroplated layers of silver.

The microscope images of electroplated layers demonstrate that the gold and silver layers were precisely and uniformly deposited on the base electrode. This shows that the gel material did not interfere with the electroplating process. The gel was easily washed away from the surface through purging with water, leaving behind a pristine plated surface. Additionally, since the gel functioned as a limiting environment for the transportation of ions, mass transfer was only restricted to diffusion, which is a crucial factor in this electrodeposition process. As a result, a current density of about 30 mA dm^−2^ (4–8 min) was found to be suitable for the electrodeposition of gold and silver layers. This amount of current density produced uniformly bright surfaces. In contrast, increasing or reducing the current density caused black surfaces and nonuniform surfaces. The black deposits (at higher current densities) could have been due to dendritic growth, which leads to a non-smooth surface finish and consequently a dark color [25]. Subsequently, a range of 600–700 mv of DC voltage between the cathode and anode was the most appropriate potential value in producing a suitable current density for the deposition of gold and silver layers. The thicknesses of the layers formed were extremely thin (a few 100 nm) due to the short duration of the electroplating and the low applied current density. The morphology of the layer formed was found to be similar to that of the underlying Cu substrate, which had the same topography. Therefore, the process produced smooth deposits similar to those made using other electroplating methods.

In order to demonstrate the formation of conformal deposits using the electroplating method and printed gels, images from layers of Cu, Ni, Au, and Ag were obtained through scanning electron microscopy (SEM). The SEM images were obtained using a TESCAN VP system, and the accelerating voltage was set at 30 kV. Figure 2d shows lowly and highly magnified (as insets) images of layers of Cu, Ni, Au, and Ag from left to right. The low-magnification SEM images show uniform and well-defined coverage of the electrode by the Ag- and Au-deposited layers. The speckle features that are visible were due to the roughness of the starting Cu substrate. The SEM images presented in the high-magnification images demonstrate that the initial surface roughness of the original Cu substrate was around one to five microns. After the deposition of the layers of Ni, Au, or Ag, the surface roughness did not change, indicating a uniform and conformal plating of the functional material over the underlying nickel base layer.

### 3.2. Parallel Electroplating of Silver and Gold

A base electrode layer made of copper polyimide foil 14 mm in length and 35 mm wide was prepared using a xurography lamination technique, as described in the experimental section. After that, nickel was coated in the base electrode through electroplating. Then, a covering layer (mask) consisting of two holes with a 1-mm radius and 7 mm apart (center to center) was laminated on the electrode. Next, a gel mixture of gold electrolytes in the amount of 10 µL was printed on one of the holes. Accordingly, the same amount of silver electrolytes mixed with gel were printed on the other hole. After that, the electrode was placed in a common working electrolyte container. Electrodeposition was performed, and layers of gold and silver were deposited on the base electrode in parallel.

**Figure 3:**
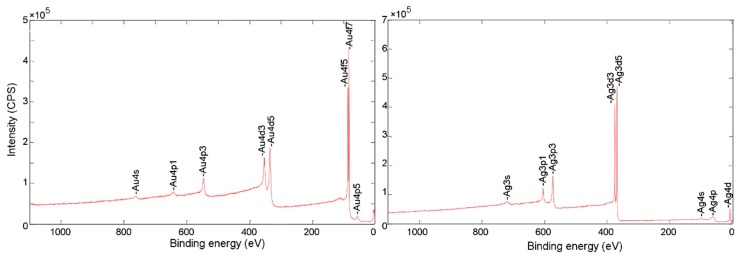
Elemental analysis of parallel electrodeposited layers of gold and silver (XPS spectra).

In order to investigate the elemental composition of the electrodeposited layers, X-ray photoelectron spectroscopy (PHI Qanutera II) was performed to comprehensively analyze the surface of the electrodeposited layers. Figure 3 shows the XPS spectra of the samples. The spectra show that silver and gold peaks were observed only in the regions that were electroplated. Therefore, electroplating by gel printing enabled the simultaneous deposition of gold and silver on the confined region without any cross-deposition. Cross-deposition was avoided by using gel as an immobilizing environment, which eliminated convective mass transport and limited transport by diffusion. Additionally, a 7-mm distance (center to center) between the two holes with a radius of 1 mm was found to be the smallest pitch size, without any cross-deposition. It is worth mentioning that holes with a radius of 1 mm were designed, as they were the minimum suitable size for elemental analysis in XPS. Designing smaller size holes (0.5 mm in radius) to place smaller droplets may reduce that distance (5 mm center to center). However, the gradual diffusion of metal ions from the gel droplets into the surrounding space can limit the ability to bring these features closer to each other. The diffusion of ions in one dimension in aqueous solution can be modeled in a simplified form [28]:

(1) $$L=\sqrt{2Dt},$$

where *L* is diffusion length, *D* is the diffusion coefficient of solute, and *t* is time. Since the diffusion coefficient of metal ions in aqueous solution is on the order of 10^−9^ m^2^ s^−1^, the ions travel about 1 mm in 5 min. It can be concluded that the diffusion of ions is a determinant for defining the minimum distance between two droplets containing different metal electrolytes. An alternate approach, such as introducing convection between the gel droplets to carry away the ions diffusing out and diluting them in the larger supporting electrolyte, can be used to mitigate the diffusional effect but still achieve closer spacing.

### 3.3. Pattern Electroplating: An Array of Dots

**Figure 4:**
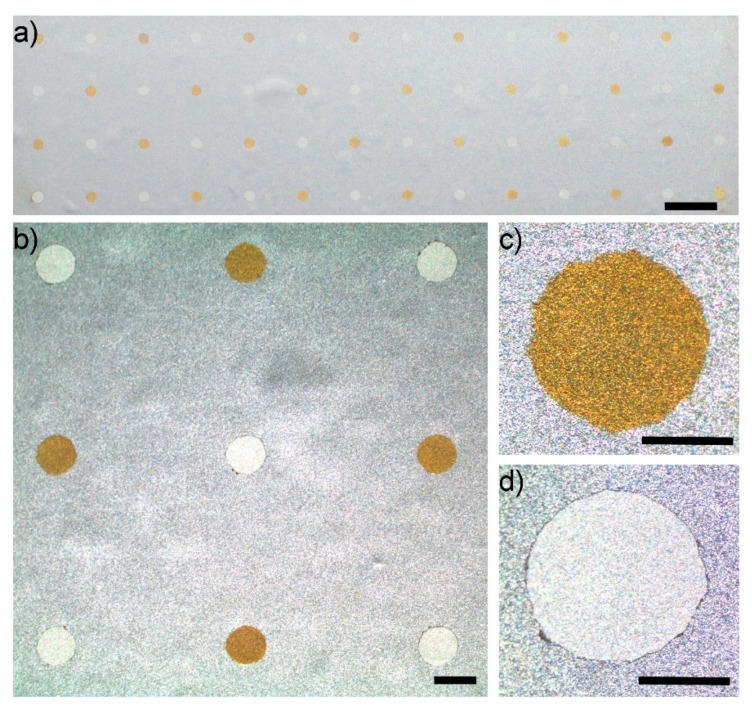
Parallel electrodeposition of gold and silver in the shape of circles on a nickel-coated substrate: (**a**) an array of 4 × 14 alternate dots (scale bar = 5 mm); (**b**) a microscope image of patterns (scale bar = 1 mm); (**c**,**d**) a microscope image of a deposited layer of (c) gold and (d) silver (scale bar = 0.5 mm).

In order to demonstrate the scalability of the technique in electroplating metals in parallel, a mask consisting of circular patterns with a radius of 500 µm placed 5 mm apart (center to center) and forming an array of 14 × 4 was fabricated using xurography. The mask was laminated on a nickel-coated copper foil with dimensions of 70 mm (L) and 45 mm (W). Further, 2-µL droplets of gel containing gold and silver electrolytes were dispensed onto alternate circular holes (patterns) on the substrate by a micropipette, and electroplating was performed as described above. After electroplating, the mask was peeled off. Figure 4a shows parallel electrodeposited layers of gold and silver on a nickel-coated substrate. In addition, a magnified image of the array is shown in Figure 4b. The microscopic images of individual circles show that layers of Ag and Au were deposited only in confined regions defined by the prepared mask (Figure 4c,d).

This result demonstrates that gel printing can be used as a comprehensive method to fabricate an electrode that is fundamentally different from conventional electroplating approaches. Conventional methods of electroplating require a set of processes that include coating and the removal of a mask that is time-consuming and expensive when multiple materials are to be electroplated. In addition, the substrate needs to be immersed in a large volume of electrolytes which is suitable for large scale production, but expensive when fabricating small-area prototype electrodes. On the other hand, electroplating with gels eliminates the need for mask coating/removal, since gel has the ability to immobilize the electrolyte solution and can be used as a direct patterning technique. This ability becomes more important when multiple materials need to be electroplated. The method of gel deposition used here was manual. However, it can be easily automated by incorporating inkjet or drop printers in order to deposit smaller volumes of gel loaded with electroplating solutions with much closer spacing. Although a xurographic patterned layer was used as a mask on the substrate, this step can also be eliminated by inkjet-printing a thin layer of insulating material as a covering layer alongside the printed electrolytes.

### 3.4. Pattern Electroplating: Lines

In order to demonstrate the ability of the gel-printing method to simultaneously electrodeposit Ag and Au in a variety of structures, masks patterned by xurography in the shape of a microfluidic channel with a width of 1 mm were used. For this purpose, thicker masks were required to carry a larger amount of plating solution uniformly across the open microfluidic channels. Therefore, masks made of polyvinyl chloride (PVC) (McMaster Carr, Elmhurst, IL, USA) with a 476-µm thickness (laminated on double-sided adhesive tape (7952, 3M™) with a 50-µm thickness) were used.

**Figure 5:**
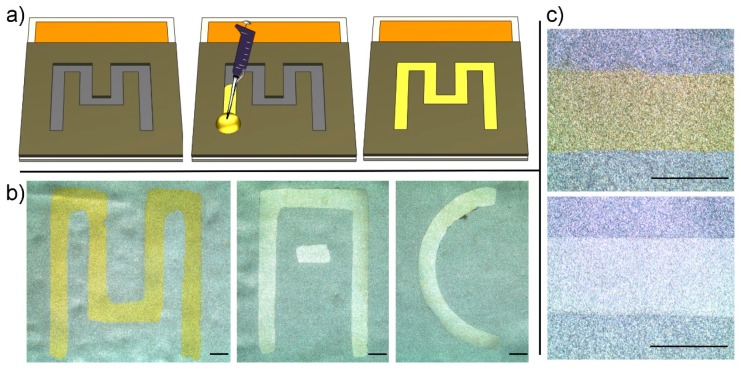
Parallel electrodeposition of gold and silver in the shape of English alphabet letters on a nickel-coated substrate. (**a**) A schematic view of the process for printing gels by using microfluidic flow (from left to right); (**b**) a microscope image of a deposited layer of gold in the shape of an “M” and a deposited layer of silver in the shape of “A” and “C”; (**c**) a microscope image of a deposited layer of gold and silver showing well-defined edges (scale bar = 1 mm).

In order to perform the experiment, a copper foil with the dimensions 30 mm (L) and 45 mm (W) was cut and coated with nickel, as described above. Then, the substrate was laminated on the prepared mask. A hot plate (VWR, Radnor, PA, USA) was used to keep the temperature of the substrate at 70 °C to prevent the gelation of the solution while the structures were filled. Between 20 and 30 µL of gel-plating solution of gold and silver were placed at the beginning of each channel, and using capillary forces, the plating solution was wicked into the open microchannels automatically (Figure 5a). Then the substrate was placed in a common working electrolyte bath, and electroplating was performed as described above. After electroplating, the mask was peeled off from the substrate.

Figure 5b shows electrodeposited layers of Ag and Au on nickel-coated substrates. As can be seen, the edges of the electrodeposited layers were well-defined compared to the nickel-coated layer (Figure 5c). Therefore, using microfluidic flow, gel solutions were patterned, and parallel electroplating of silver and gold was performed. Additionally, the use of capillary wicking eliminated the manual dispensing of gels over entire patterns, which facilitated the printing process. However, for longer distance patterns, capillary flow slows down toward the end of the pattern. In that case, modifying the channel design, such as using slightly conical shape channels or placing a temporary sealing layer on top of the open channel, will enhance the capillary pumping [29,30].

### 3.5. Electrochemical Sensing

Electrochemical sensors have gained increasing attention for chemical [5] and biochemical analyses [31] in microfluidics. These methods are highly sensitive and selective, and they use a very small amount of electrical power. These significant characteristics have made electrochemical sensing an ideal choice for miniaturization. However, the conventional fabrication techniques for electrochemical microfluidic sensors are complicated and tedious. In addition, they require a cleanroom and high-cost facilities.

A microfluidic device consisting of an electrochemical sensor and a microchannel was fabricated to demonstrate the capability of the developed technique in the fabrication of high-quality electrodes. In this device, an array of three electrodes that were 2.5 mm wide and 20 mm in length with a gap size of 20 mm was fabricated from copper polyimide film using xurography. The electrodes, including working, counter-, and reference electrodes, were initially coated with nickel, as described above. Next, a layer made of one-sided adhesive tape (9795MP-3M™) consisting of three 1-mm (radius) holes was laminated on the electrodes. These holes defined the contact area between the electrodes and the analyte flow. Next, gel droplets of electrolytes of gold and silver were printed on the side electrodes and on the middle one, respectively. After that, electrodeposition was applied as described in the experimental section.

**Figure 6:**
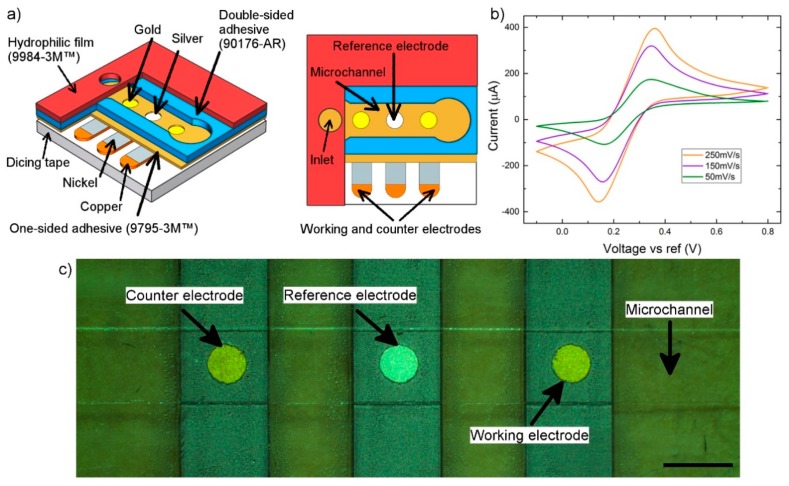
An integrated microfluidic sensor. (**a**) Layer-by-layer structure of the microfluidic device; (**b**) cyclic voltammogram of 100 mM ferricyanide/ferrocyanide; (**c**) microchannel and three-electrode system in a microfluidic sensor (scale bar = 2 mm).

After that, a layer of double-sided adhesive tape (90176, Adhesive Research, Glen Rock, PA, USA) consisting of a microfluidic channel that was 2 mm wide, 20 mm long, and 89 µm in height was added. Finally, a layer of hydrophilic polyester film (9984, 3M™) was laminated on the device. This layer consisted of a circular inlet and outlet of the liquid channel with a radius of 1 mm (Figure 6a). This layer automatically wicked the microchannel using capillary forces that eliminated the need for any equipment for the sample injection. After assembly, 20 µL of 1-M potassium chloride (Caledon Laboratories Ltd., Georgetown, ON, Canada) was injected at the inlet of the microchannel. The solution filled the channel automatically due to the hydrophilic nature of the channel. After that, 2 V of DC as an anode was applied for 30 s to the silver electrode to change its surface into silver chloride. Therefore, an electrochemical microfluidic sensor consisting of a three-electrode system, including a silver/silver chloride reference electrode, a gold working electrode, and a counterelectrode, was fabricated (Figure 6c).

In addition, 20 µL of 100-mM potassium hexacyanoferrate (Sigma-Aldrich, St. Louis, MO, USA) was injected at the inlet of the microfluidic device. Due to the hydrophilicity of the microchannel, the sample filled the microchannel automatically without the need for any excess action. After that, a potentiostat (EmStat2, PalmSense, Houten, The Netherlands) was used to apply cyclic voltammetry (CV) to the sample. Different scan rates were selected, and data were acquired accordingly (Figure 6b). The results revealed that the current at the cathodic and anodic peaks of the ferricyanide/ferrocyanide redox couple was proportional to the scan rate.

The parallel electroplating technique combined with the xurography lamination technique implemented the integration of high-quality electrode sensors into a microfluidic device in a fast and facile way. The entire process, including cutting the electrodes and layers, gel printing, electrodeposition, and lamination, was performed in less than 30 min. This time is significantly lower than that required by standard microfluidic sensor fabrication techniques, which need multiple sequential steps to deposit and remove each material. In addition, the gel-printing method enabled us to electrodeposit silver and gold simultaneously in a single process on a single layer. Automatic filling of the device using a hydrophilic film made this sensor suitable for end users. Further, the small amount of electroplating solution and the small number of copper and plastic films and facilities used in this approach make the entire process inexpensive and suitable for commercialization.

## 4. Conclusions

In order to reduce the process steps for the fabrication of electrochemical sensors, a novel method for the parallel electroplating of noble metals such as gold and silver was introduced. In this approach, we used gels to immobilize and pattern electroplating electrolytes on a substrate surface. Using this method, a significant volume of electrolytes was conserved, since the method only used droplets of gels. In addition, significant amount of time was saved, as multiple metals were electroplated in a single step. We showed the reliability of this method through the parallel electrodeposition of gold and silver in the form of arrays of dots and lines. We also showed an application of this technique in which an integrated three-electrode microfluidic device performed electrochemical sensing. In conclusion, this parallel electroplating technique can be broadly used for the fabrication of analytical devices in a wide range of bioanalytical and environmental applications, such as diagnostics and pollution monitoring. For the further development of this technique, the electroplating of other functional metals such as palladium, platinum, copper, and tin, which have been extensively used in electrochemical sensors, can be investigated.
